# Photodynamic therapy-generated vaccines: relevance of tumour cell death expression

**DOI:** 10.1038/sj.bjc.6604059

**Published:** 2007-10-30

**Authors:** M Korbelik, B Stott, J Sun

**Affiliations:** 1 Department of Cancer Imaging, British Columbia Cancer Agency, Vancouver, British Columbia, Canada

**Keywords:** cancer vaccine, photodynamic therapy, degranulating CD8^+^ cells, cell death

## Abstract

Recent investigations have established that tumour cells treated *in vitro* by photodynamic therapy (PDT) can be used for generating potent vaccines against cancers of the same origin. In the present study, cancer vaccines were prepared by treating mouse SCCVII squamous cell carcinoma cells with photosensitiser chlorin e6-based PDT and used against poorly immunogenic SCCVII tumours growing in syngeneic immunocompetent mice. The vaccine potency increased when cells were post-incubated in culture after PDT treatment for 16 h before they were injected into tumour-bearing mice. Interfering with surface expression of phosphatidylserine (annexin V treatment) and apoptosis (caspase inhibitor treatment) demonstrated that this post-incubation effect is affiliated with the expression of changes associated with vaccine cell death. The cured mice acquired resistance to re-challenge with the same tumour, while the engagement of cytotoxic T lymphocytes was demonstrated by detection of high numbers of degranulating CD8^+^ cells in vaccinated tumours. The vaccines prepared from *ex vivo* PDT-treated SCCVII tumour tissue were also highly effective, implying that surgically removed tumour tissue can be directly used for PDT vaccines. This opens attractive prospects for employing PDT vaccines tailored for individual patients targeting specific antigens of the patient's tumour.

Although photodynamic therapy (PDT) is established for clinical treatment of neoplastic and other lesions (Dougherty *et al*, 1998; Brown *et al*, 2004; Huang, 2005), this modality is under continued investigation for improved efficacy and expanded use. The destruction of targeted lesions by PDT results from localised production of reactive oxygen species mediated by drugs (photosensitisers) capable of capturing the energy of light and transferring it to molecular oxygen (Henderson and Dougherty, 1992; Dougherty *et al*, 1998). Unlike immunologically silent genotoxic damage produced by radiotherapy and chemotherapy, photooxidative cytotoxic lesions generated by PDT are extranuclear and result in a rapid cell death that alerts host's immune surveillance elements (Castano *et al*, 2006; Gollnick *et al*, 2006; Korbelik, 2006). Hence, tumour PDT induces a strong host response mediated by innate immune system and characterised by overt inflammatory and acute phase responses that culminates in the acquirement of adaptive immunity recognising the treated tumour as its target (Korbelik, 2006).

A particularly interesting exploitation of the immune-activating capacity of PDT is the development of PDT-generated vaccines. The preparation designed by Gollnick *et al* (2002), made of the lysates of mouse tumour cells treated *in vitro* by PDT, was shown to effectively protect against challenge of mice with the same tumour cells. The authors showed that the action of this prophylactic vaccine is associated with phenotypic and functional maturation of dendritic cells and the induction of a cytotoxic T-cell response, and is based on tumour-specific immune response (ineffective against mismatched tumour types). Therapeutic PDT vaccine preparations based on whole tumour cells were developed in our laboratory (Korbelik and Cecic, 2003; Korbelik and Sun, 2006). In this case, vaccination with *in vitro* expanded and PDT-treated mouse squamous cell carcinoma SCCVII cells of mice-bearing established subcutaneous SCCVII tumours produced growth retardation and cures of these poorly immunogenic lesions. The therapeutic effect was also shown to be based on the elicited tumour-specific adaptive immune response (Korbelik and Sun, 2006). The distinctive advantage in using PDT for the generation of cancer vaccines is evidenced by the fact that this cannot be rivalled by comparable treatments with tumour cells exposed to X-rays, UV, hyperthermia or their freeze-thaw lysates (Gollnick *et al*, 2002; Korbelik and Sun, 2006). This advantage, as well as more favourable perspectives compared to other polyvalent vaccination strategies like whole cell RNA-mediated transfection (Heisler *et al*, 2001), appears to be imparted by a combination of highly amplified immunogenicity rendered through photooxidative alterations of tumour proteins and delicate balance of apoptotic and necrotic death induced in vaccine cancer cells (as will be further circumstantiated in the present work).

Despite increasing numbers of identified tumour-specific antigens, there are clear advantages of whole-cell/polypeptide vaccination over targeting specific epitopes. The polyvalent vaccines, such as autologous whole-cell vaccines represented by PDT vaccines, secure greater coverage of potential/diverse tumour antigens (even if most of them are unknown) and include the necessary determinants for helper T cells (Copier and Dalgleish, 2006; Emens, 2006). Autologous whole-cell vaccines are optimally conditioned to express antigens in patient-matched MHC and are less likely to encounter ‘tumour escape’ by downregulation of antigen expression (Khong and Restifo, 2002; Emens, 2006). Clinically significant benefit using such autologous approach has been established from randomised trials for colorectal and renal cancer (Uyl-de Groot *et al*, 2005; Zhou *et al*, 2005). The possibility of tailoring individual vaccines matching the antigenic profile of patient's tumour (Hoos *et al*, 2004; Lewis, 2004) is illustrated in the present report that also describes further advances in improving the potency of PDT vaccines and gives new insights on the underlying mechanisms.

## MATERIALS AND METHODS

### Tumour model

Mouse squamous cell carcinoma SCCVII, recognised as an excellent model for poorly immunogenic head and neck cancer of spontaneous origin (Khurana *et al*, 2001), was maintained *in vivo* by serial transplantation into syngeneic C3H/HeN mice. Experimental tumours were initiated by injecting subcutaneously 1 million cells in the lower dorsal region of 7–9 weeks old female mice. The cultures of SCCVII cells *in vitro* were grown in Alpha Minimal Essential Medium (Sigma Chemical Co., St Louis, MO, USA) supplemented with 10% fetal bovine serum (HyClone Laboratories Inc., Logan, UT, USA). The vaccination was carried out as a single peritumoral injection (unless stated differently), and was performed at 6 days post-tumour implant when these lesions reached 5 mm in largest diameter. After vaccination, tumour sizes were monitored by measuring the lesion's three orthogonal diameters with a caliper. The follow-up period after vaccination lasted until the tumours in control groups became too large (>12 mm largest diameter) for keeping the mice due to ethical considerations. In cases of vaccine-induced complete tumour regression, the mice were kept under observation for up to 90 days and those remaining tumour-free at that time were declared cured. Each treatment group consisted of six mice. The protocols used with mice were approved by the Animal Care Committee of the University of British Columbia.

### Vaccine generation

In initial experiments, vaccine generation procedure described in a previous report (Korbelik and Sun, 2006) was followed with the exception of using photosensitiser chlorin e6 (Ce6, Frontier Scientific Inc., Logan, UT, USA); this is referred to as ‘standard vaccine protocol’. Briefly, required numbers of SCCVII cells were incubated with ce6 (0.5 or 2.5 *μ*g ml^−1^) in serum-free medium for 30 min at 37°C, washed and exposed to 1 J cm^−2^ (15 mW cm^−2^) of 665±10 nm light, then treated with X-rays (60 Gy), and immediately thereafter injected peritumorally (2 × 10^7^ cells per mouse, 0.2 ml volume). The light source used was an FB-QTH-3 high throughput illuminator (Sciencetech Inc., London, Ontario, Canada) equipped with a 150 W QTH lamp and suitable interference filter. The X-ray source was Philips RT 250 (250 kV, 0.5 mm Cu, dose rate 3.26 Gy min^−1^). Control groups included tumour-bearing mice injected with the same number of SCCVII cells that received only X-ray treatment (no PDT).

In the course of this study a new protocol was developed referred to as ‘post-incubation vaccine protocol’. It differs from the standard vaccine protocol only in including a 16-h (overnight) incubation of SCCVII cells at 37°C after they were treated with PDT light; the cells were kept in a specially enriched serum- and protein-free medium (S8284, Sigma) distributed at 4 × 10^7^ cells per 175 cm^2^ T-flask with 20 ml medium. The cells were then concentrated for X-ray treatment and injection into mice. The medium in which the cells were ‘post-incubated’ was in some cases also collected, then concentrated using molecular filters (Centricon Plus-80, 30,00 MWCO, by Millipore Corporation, Bedford, MA, USA), and included together with the vaccine cells into the 0.2-ml volume injected into mice.

To investigate the role of apoptotic cell death in PDT vaccine cells, apoptosis blocking agent caspase-3 inhibitor benzyloxycarbonyl-Asp(OMe)-Glu(Ome)-Val-Asp(OMe)-fluoromethyl ketone (Z-DEVD, purchased from Enzyme Systems Products Inc., Livermore, CA, USA) was added to the medium at the concentration of 33.4 *μ*g ml^−1^ (50 *μ*M) at the onset of 16-h post-incubation of PDT-treated SCCVII cells. Alternatively, the cells were post-incubated without Z-DEVD but were exposed immediately thereafter to annexin V (BioVision Research Products, Mountain View, CA, USA) at the concentration of 0.2 mg ml^−1^ in annexin V binding buffer (PharMingen BD Biosciences, Mississauga, Ontario, Canada) for 30 min at room temperature. The cells were then collected, concentrated for X-ray treatment and injected as PDT vaccine.

For preparing the PDT vaccine from tumour brei, tissue of growing untreated tumours was finely minced using a pair of scalpels and frozen until use in liquid nitrogen. Freshly thawed brei was incubated *ex vivo* with ce6 (0.5 *μ*g ml^−1^) and further processed according to the post-incubation vaccine protocol. The vaccine injection volume (200 *μ*l) contained 170 *μ*l of wet brei plus 30 *μ*l of phosphate-buffered saline (the highest brei concentration that can pass through the needle).

### Flow cytometry

The procedure used was recently described in detail (Korbelik and Cooper, 2007). Briefly, tumours were excised at 3 days after vaccination, weighed, enzymatically disaggregated, and the obtained single cell suspensions counted and subjected to surface staining with antibodies for three-colour flow cytometry. The antibodies included FITC-conjugated rat anti-mouse CD107a and PE-conjugated rat anti-mouse CD8 (both from Santa Cruz Biotechnology Inc., Santa Cruz, CA, USA), and biotin-labelled rat anti-mouse CD11b (Mac-1, eBioscience Inc., San Diego, CA, USA). The latter antibody was detected by streptavidin-conjugated Cy-Chrome (PharMingen BD Biosciences). The isotype control for anti-CD107a was rat anti-mouse IgG_2a_ (eBioscience). The samples were analysed with a Coulter Epics Elite ESP (Coulter Electronics, Hialeah, FL, USA) including 20 000 cells for each test. Degranulating cytotoxic T lymphocytes were identified as CD8^+^CD107a^+^ cells from populations gated negative for myeloid marker Mac-1.

Additional flow cytometry analysis was carried out for determining the percentage of apoptotic and necrotic cells that appear following the post-incubation protocol for PDT vaccine generation. For this purpose the cells were stained with FITC-conjugated annexin V followed by PE-conjugated rabbit monoclonal antibody against active caspase-3 diluted in Perm/Wash buffer (all from PharMingen). Apoptotic cells were identified as positively stained with both antibodies, while necrotic cells were positively stained only with annexin V.

### Statistical analysis

Tumour growth-inhibition results and mouse survival results were statistically evaluated using log-rank test. The differences between means of the data from remaining results were analysed with Mann–Whitney test. Significance level of 5% was set as a threshold for determining if the groups were statistically different.

## RESULTS

**Figure 1:**
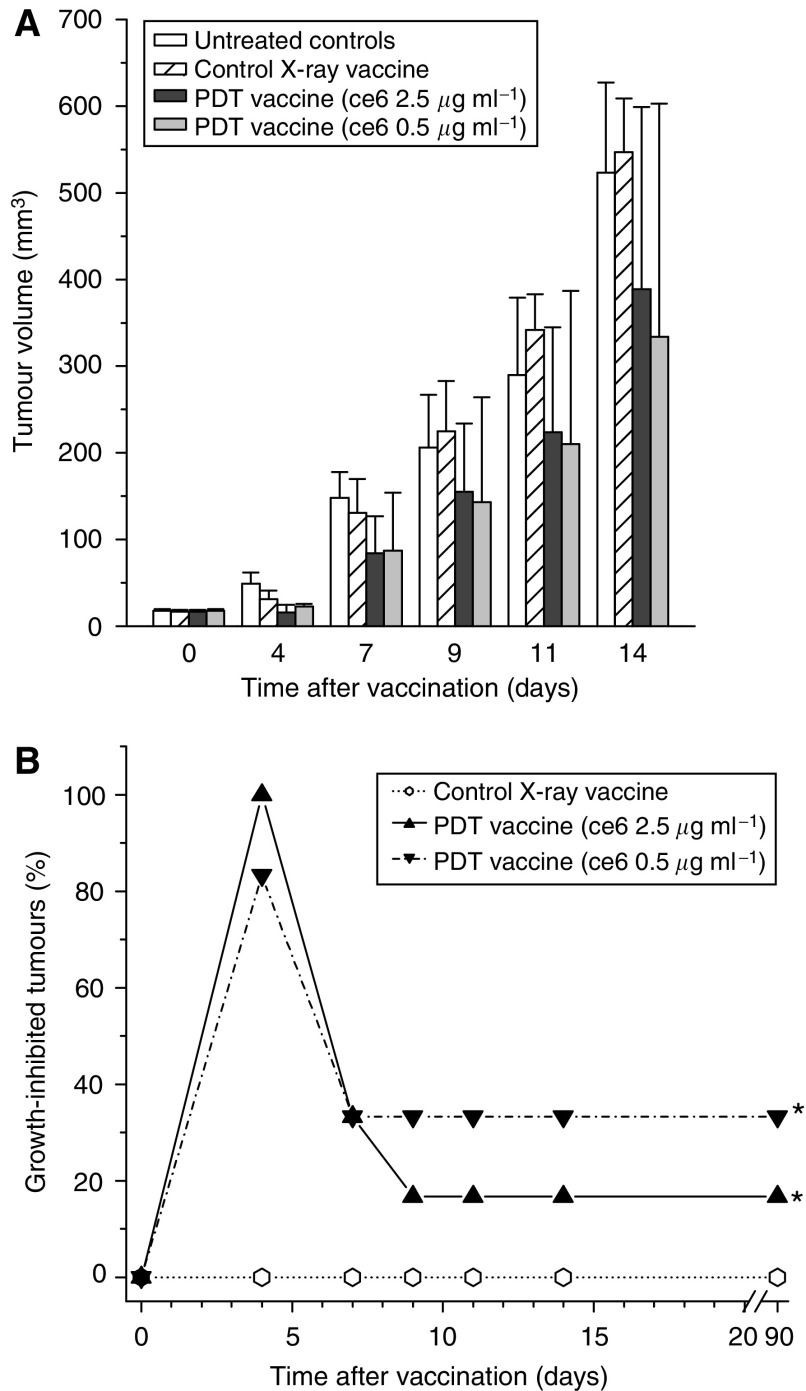
The effect of photodynamic therapy (PDT)-generated vaccine prepared using photosensitiser ce6 on growth of established SCCVII tumours. The vaccine was prepared by standard vaccine protocol (see Materials and Methods section) involving the incubation of SCCVII cells with ce6 (0.5 or 2.5 *μ*g ml^−1^) for 30 min, then illumination (1 J cm^−2^), and exposure to X-rays (60 Gy) followed immediately by their injection in SCCVII tumour-bearing mice (2 × 10^7^ cells per mouse, peritumorally). The therapy response was monitored by tumour size measurement and is presented as (**A**) means for tumour volume values plus s.d., and (**B**) percentage of growth-inhibited tumours (smaller than the means minus two-fold s.d. of unvaccinated control group). The controls are showing growth of vaccine untreated and X-ray only vaccine treated tumours. Each treatment group consisted of six mice. ^*^Indicates statistical significance (*P*<0.05) for the difference in tumour growth compared to untreated controls.

The effectiveness of PDT vaccines prepared by employing photosensitiser ce6 (not tested before for this application) against subcutaneous SCCVII tumours growing in C3H/HeN mice is shown in Figure 1. The vaccines were prepared by exposing *in vitro* expanded SCCVII cells to either 0.5 or 2.5 *μ*g ml^−1^ ce6 for 30 min in serum-free medium followed by treatment with 1 J cm^−2^ of 665±10 nm light. The PDT dose with the lower ce6 concentration kills around ⅔ of cells, while treatment involving the higher ce6 concentration is supralethal (data not shown). Immediately thereafter, the cells were concentrated, treated with a lethal dose of X-rays and delivered by perilesional injection (2 × 10^7^ per mouse) to mice-bearing established SCCVII tumours. Except for the photosensitiser, this procedure followed the protocol optimised in our previous studies (Korbelik and Sun, 2006). Also included in this experiment were tumour-bearing mice serving as vaccine-untreated controls (received only the perilesional injection of the same volume of saline) and X-ray only controls (received the perilesional injection of the same number of cells that were treated only with X-rays). The measurement of tumour sizes after vaccination revealed that the tumours in both PDT vaccine groups remained on average smaller than the control tumours, while there was no obvious difference in sizes of tumours in the group receiving X-ray only treated cells compared to the control group (Figure 1A). Statistically significant inhibition of tumour growth by both PDT vaccines was evident at 4 days post-vaccination, but the statistical significance could not be reached at the later time points due to large values of s.d. with the PDT vaccine groups. These s.d. were large because the PDT vaccines had a strong impact on some and limited effect on other tumours within the same treatment group. In such situation, instead of averaging the size for the whole treatment group it is more informative to express the result by listing the percentage of tumours that were significantly growth-inhibited. The results of such analysis, with tumours smaller than the means minus two-fold s.d. of the control group qualifying as growth-inhibited, are depicted in Figure 1B. With both PDT vaccine groups there was a significant therapeutic effect (*P*<0.05, statistically evaluated by log-rank test). This was manifested as an initial growth-inhibitory effect including most or all tumours and persisting in the one-third of tumours with the PDT vaccine group based on the lower ce6 dose (0.5 *μ*g ml^−1^) that appeared more effective than the PDT vaccine using the higher ce6 dose (although statistically the difference was not resolved). In this experiment, the tumours that qualified as growth-inhibited at 9 days post-vaccination become impalpable and showed no signs of recurrence up to 90 days post-vaccination which categorises them as cured. On day 100 post-vaccination, these cured mice were re-challenged by an inoculum of 1 × 10^6^ SCCVII cells. Subsequent monitoring showed no tumour growth, which demonstrates that the mice acquired resistance against the vaccinated tumour.

**Figure 2:**
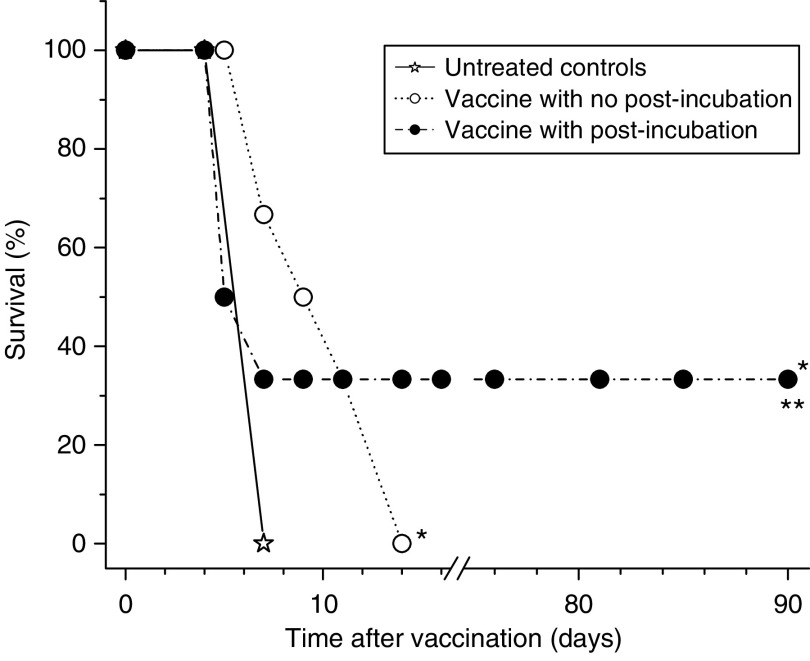
Comparison of the effects of photodynamic therapy (PDT) vaccines prepared by standard and post-incubation protocols. The preparation of PDT vaccine was based on the treatment of SCCVII cells with ce6 (0.5 *μ*g ml^−1^) as described for Figure 1, except that 16-h post PDT incubation was or was not also included (see Materials and Methods section). The response of SCCVII tumours after peritumoral injection of 2 × 10^7^ cells per mouse is presented as survival of mice (terminating when tumours reached 100 mm^3^). Each treatment group consisted of six mice. ^*^Indicates statistical significance (*P*<0.05) for the difference in survival compared to untreated controls; ^**^Indicates statistical significance (*P*<0.05) for the difference in survival compared to the vaccine with no post-incubation treatment group.

The ce6 dose of 0.5 *μ*g ml^−1^ was chosen for PDT vaccines in the remaining experiments of this study. Examined next was the possibility that the effectiveness of PDT vaccine can be augmented by keeping the vaccine cells after PDT treatment under culture conditions at 37°C to allow the progression of induced molecular/biological events that may prove relevant for the vaccine's action. Hence, tumour-bearing mice received in the next experiment either the standard PDT vaccine (cells used immediately after PDT treatment) or PDT vaccine consisting of cells incubated after PDT treatment for 16 h at growth conditions before harvested for injection (post-incubation vaccine protocol). The response (depicted as survival, based on the percentage of mice whose tumours remained smaller than 100 mm^3^) is shown in Figure 2. It can be seen that both vaccination protocols significantly prolonged the survival of tumour-bearing mice, but the therapeutic benefit was significantly better after the post-incubation vaccine protocol and produced tumour cures with one-third of treated mice. Hence, this more effective vaccine protocol was adopted for the remaining experiments in this study.

**Figure 3:**
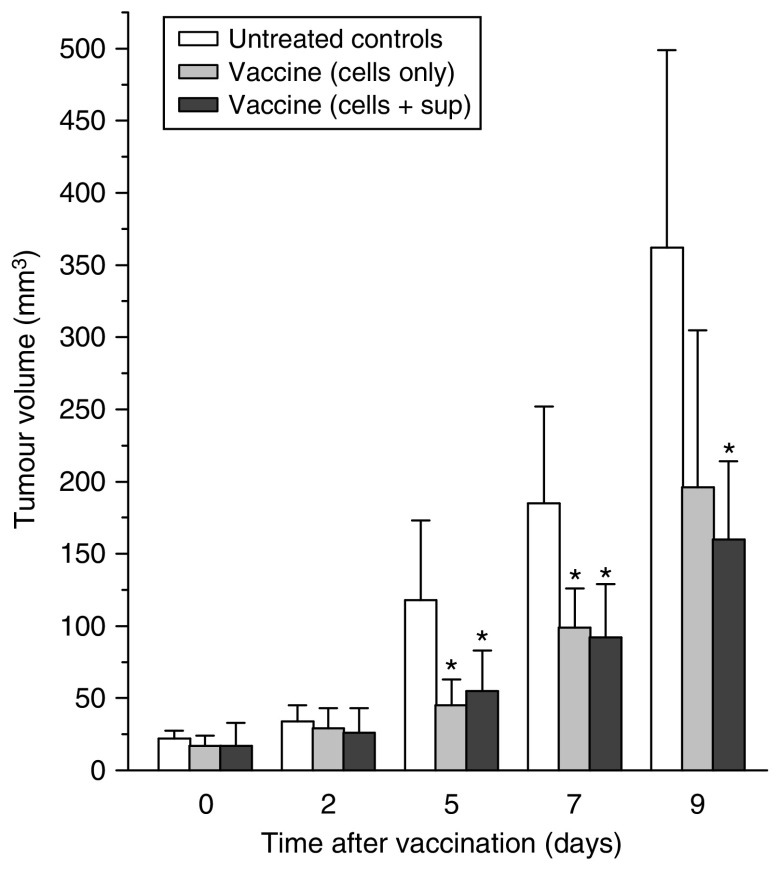
Testing of photodynamic therapy (PDT) vaccine cell supernatant. The PDT vaccine was prepared using the post-incubation protocol described for Figure 2. In addition to vaccine cells, the vaccine injection in one group of mice included concentrated post-incubation medium supernatants. The results are presented as histograms depicting means for tumour volume plus s.d. Each treatment group consisted of six mice. ^*^Indicates statistical significance (*P*<0.05) for the difference in tumour size compared to the untreated controls at the same time interval.

In the next experiment, one group of tumour-bearing mice received the vaccine that contained not only PDT-treated cells collected after the post-incubation protocol but also the concentrated supernatant (medium) collected from the same cultures. At 5, 7 and 9 days post-vaccination the tumours in this group were significantly smaller than the tumours in unvaccinated control group but their size was not significantly different than the size of tumours in the group that received the PDT-treated and post-incubated cells without the supernatant (Figure 3). On the basis of these results, it appears that adding the supernatant concentrate to the cells affords no additional benefits. A detectable effect was also observed with the supernatant concentrate used alone, but it was inferior to that obtained with cell-based vaccines (data not shown). Aliquots of these cells taken after the post-incubation PDT vaccine protocol were analysed by flow cytometry, which showed that 40–50% of them were apoptotic (positively stained with antibody against active caspase-3 and annexin V) and around 10% were necrotic (stained with annexin V but not positive with anti-active caspase 3).

**Figure 4:**
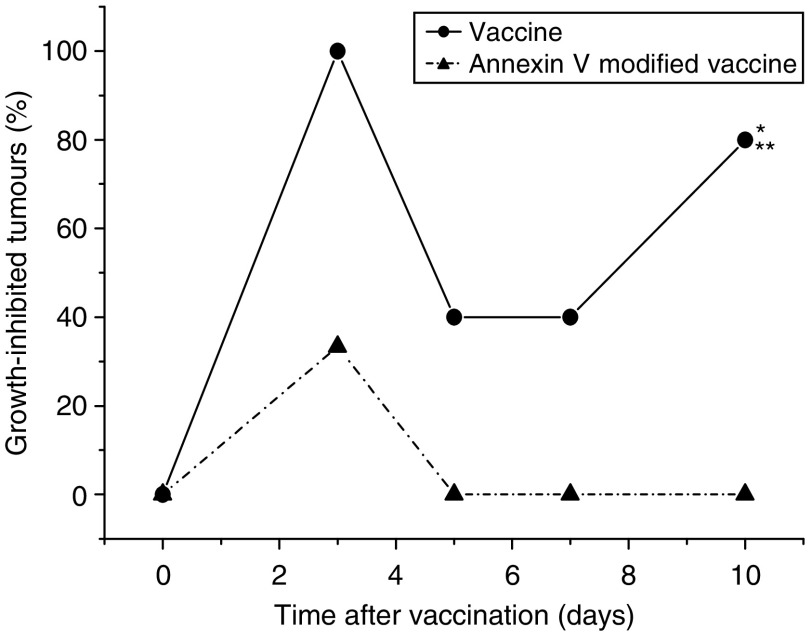
The effect of exposing photodynamic therapy (PDT) vaccine cells to annexin V. Mice-bearing SCCVII tumours received peritumoral injection of PDT vaccine consisting of SCCVII cells treated by the post-incubation protocol described for Figure 2, but including in one group also a 30-min exposure of cells to annexin V (0.2 mg ml^−1^, see Materials and Methods section). The therapy response was monitored by tumour size measurement and is presented as percentage of growth-inhibited tumours (same as Figure 1B). Each treatment group consisted of six mice. ^*^Indicates statistical significance (*P*<0.05) for the difference in tumour growth compared to untreated controls; ^**^Indicates statistical significance (*P*<0.05) for the difference in tumour growth compared to the annexin V-modified vaccine treatment group.

**Figure 5:**
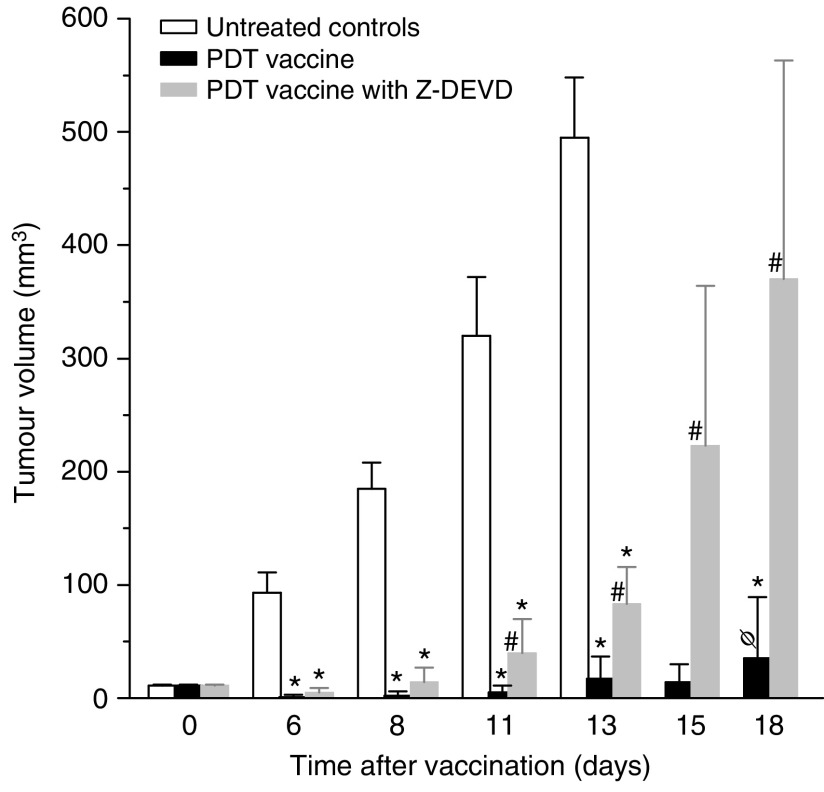
The effect of apoptosis inhibition in photodynamic therapy (PDT) vaccine cells. Mice-bearing SCCVII tumours received peritumoral injection of PDT vaccine consisting of SCCVII cells treated by the post-incubation protocol described for Figure 2, except that Z-DEVD (33.4 *μ*g ml^−1^) was present in the post-incubation medium for one group. The therapy response was monitored by tumour size measurement and is presented as the means for tumour volume values plus s.d. Each treatment group consisted of six mice. ^*^Indicates statistical significance (*P*<0.05) for the difference in tumour size compared to the untreated controls at the same time interval; ^#^Indicates statistical significance (*P*<0.05) for the difference in tumour size compared to the vaccine without Z-DEVD treatment group at the same time interval. Φ=66.7% were tumour free at 90 days post-vaccination.

Exposure of PDT vaccine cells after the 16-h post-incubation to annexin V (known to bind phosphatidylserine) dramatically affected the efficacy of the vaccine. While PDT vaccine not involving annexin V exposure strongly inhibited tumour growth (statistically significant effect, *P*<0.05), this effect was almost completely abolished in mice receiving annexin V modified vaccine (Figure 4). An assumption that phosphatidylserine as a cell death marker is relevant in this situation was supported by the results of experiments in which Z-DEVD, inhibitor of apoptosis, was included in the cell medium during the 16-h post-incubation of PDT vaccine cells. Compared to very effective tumour growth inhibition by PDT vaccine not involving Z-DEVD treatment (statistically significant reduction in tumour sizes from day 6 onwards) eventually manifested as tumour cures in two-thirds of treated mice, the effect on tumour growth of Z-DEVD modified PDT vaccine was greatly diminished (statistically greater tumour sizes for days 11–18 compared to PDT vaccine only group) and produced no tumour cures (Figure 5).

**Figure 6:**
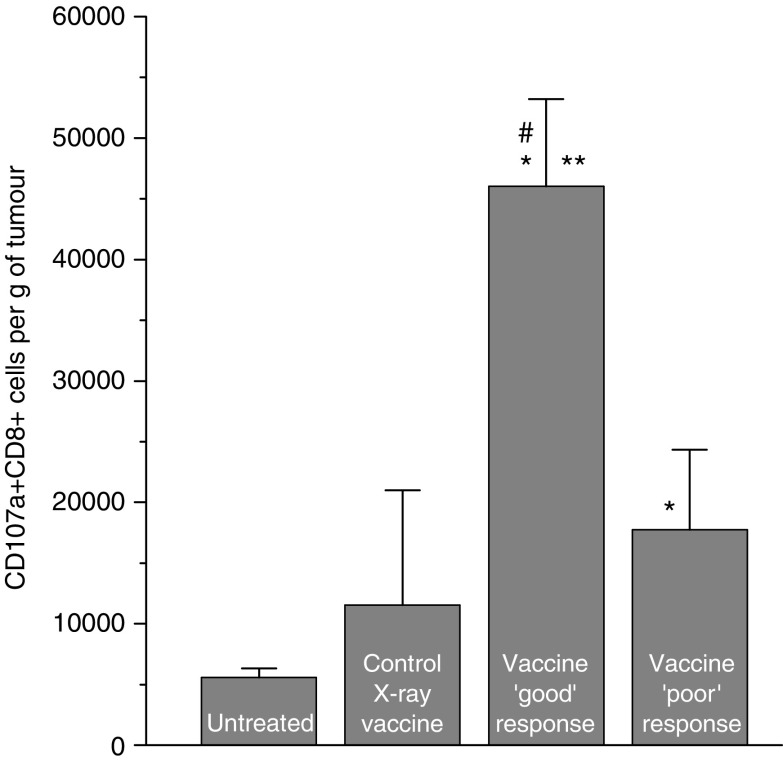
Detection of degranulating cytotoxic T lymphocytes in tumours treated by photodynamic therapy (PDT) vaccine. Mice-bearing SCCVII tumours received peritumoral injection of PDT vaccine (SCCVII cells treated by the post-incubation protocol described for Figure 2). The tumours were excised 3 days later and disaggregated into single cell suspensions that were stained with antibodies against mouse CD8, CD11b and CD107a antigens, and analysed by flow cytometry. Degranulating cytotoxic T lymphocytes were identified as CD8^+^CD107a^+^ cells in populations gated negative for staining with myeloid marker CD11b. Samples from tumours that regressed by the time of excision (good early responders) and those from tumours that continued growing (poor early responders) were analysed separately. Also included were samples from control sham-vaccinated tumours (saline injected) and from tumours injected with SCCVII cells treated only with X-rays. The experimental groups consisted of four or five mice. Bars are s.d. ^*^Indicates statistical significance (*P*<0.05) for the difference in the number of CD8^+^CD107a^+^ cells compared to the untreated control group; ^**^Indicates statistical significance (*P*<0.05) for the difference in the number of CD8^+^CD107a^+^ cells compared to the poor early responders group; ^#^Indicates statistical significance (*P*<0.05) for the difference in the number of CD8^+^CD107a^+^ cells compared to the control X-ray vaccine group.

The results of previous investigations of PDT vaccines suggest that their effect is based on the action of tumour-specific cytotoxic T lymphocytes (Gollnick *et al*, 2002; Korbelik and Sun, 2006). To obtain direct evidence for this, tumours were collected at 3 days after vaccination (PDT vaccine generated by the post-incubation protocol followed by standard X-ray dose) and disaggregated for obtaining single cell suspensions for flow cytometry analysis. For detecting degranulating cytotoxic T cells (which happens only when these immune effectors encounter their specific targets) staining was performed with anti-mouse CD107a antibody. Lysosome-associated membrane protein-1 (designated CD107a) is expressed on the surface of these cells only after exocytosis of their granzyme and perforin-rich granules that happens while engaging in the attack of specific targets in an antigen-specific manner (Betts *et al*, 2003; Burkett *et al*, 2005). The cells of interest were identified as positively stained for CD107a and CD8 antigens and negatively stained for myeloid marker Mac-1. Separately analysed were samples from PDT vaccine good early responders (tumours regressed) and poor early responders (tumours continued growing). Compared to control unvaccinated tumours, ‘good’ responders contained almost 10-fold higher number of degranulating CD8 cells at the lesion site, while the number of these cells in ‘poor’ responders was significantly lower (although still significantly elevated compared to the control tumours) (Figure 6). An increase in average number of CD107a^+^CD8^+^ cells was also registered in tumours that received control vaccination (cells treated with X-rays only), but the difference compared to the unvaccinated controls was not statistically significant.

**Figure 7:**
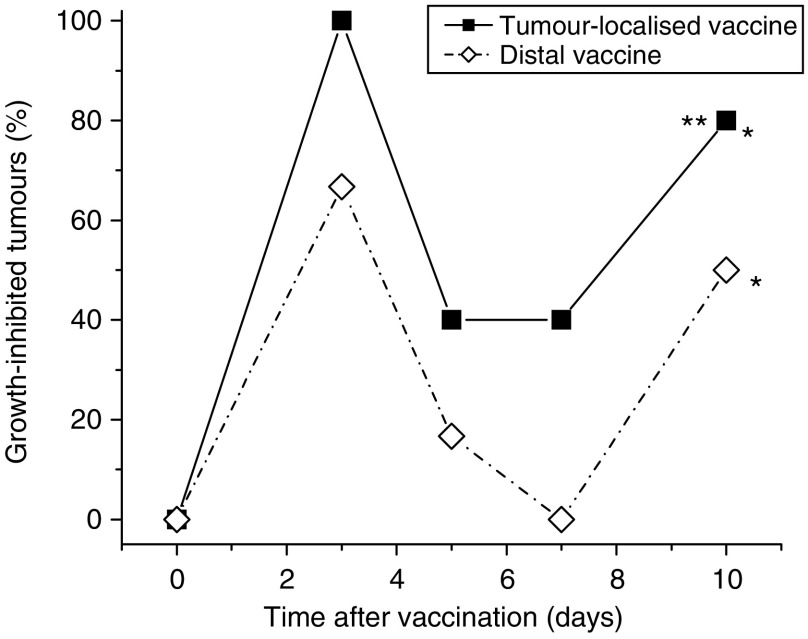
Comparison of the effects of locally (peritumorally) or distally administered photodynamic therapy (PDT) vaccines. Mice-bearing SCCVII tumours received PDT vaccine (prepared by the post-incubation protocol described for Figure 2) injected either peritumorally or at a distal location (subcutaneously at dorsal neck site). Tumour responses are presented as percentage of growth-inhibited tumours (same as in Figure 1B). Each treatment group consisted of six mice. ^*^Indicates statistical significance (*P*<0.05) for the difference in tumour growth compared to the untreated controls group; ^**^Indicates statistical significance for the difference in tumour growth compared to the distal vaccine treatment group.

To test the relevance of the distance of vaccination site from the targeted tumour, the effects of PDT vaccine delivered locally (peritumoral injection, lower sacral region) and at a distant site (subcutaneous injection at dorsal neck site) were compared in the same experiment. The results, showing the percentage of growth-inhibited tumours during the post-vaccination follow-up, are depicted in Figure 7. It is evident that both vaccine treatments resulted in a pronounced retardation of tumour growth. A strong growth inhibition was evident already at 3 days after injection and expressed prominently again after temporal diminution at 5- and 7-day time points. The latter apparent transient alleviation in the vaccine effect coincides with the switch to more rapid growth kinetics of control tumours (boosted by blood vessel build-up) that can be delayed in individual cases; the impact of such growth pattern with some untreated tumours was not always evident but is also detectable in Figure 4. Although statistical difference compared to the unvaccinated control group was attained by both tumour-localised and distal vaccine treatments, the therapeutic effect of locally injected vaccine was significantly better than the distal.

**Figure 8:**
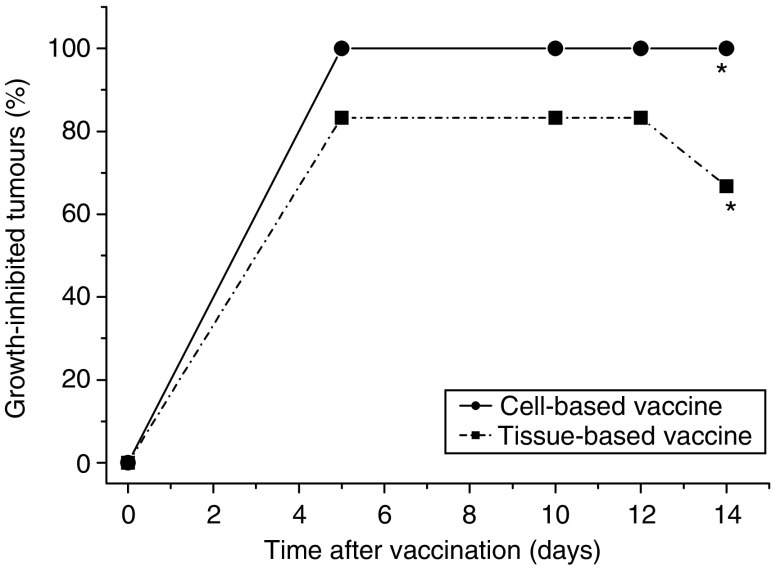
The effect of photodynamic therapy (PDT) vaccine prepared directly from tumour tissue. Mice-bearing SCCVII tumours received a peritumoral injection of PDT vaccine prepared either from SCCVII cells or from SCCVII tumour tissue brei using in both cases the post-incubation protocol described for Figure 2). Tumour responses are presented as percentage of growth-inhibited tumours (same as in Figure 1B). Each treatment group consisted of six mice. ^*^Indicates statistical significance (*P*<0.05) for the difference in tumour growth compared to the untreated controls group.

To examine whether tumour tissue can be used directly for PDT vaccine, brei (minced tissue) of SCCVII tumours (freshly thawed from storage in liquid nitrogen) instead of SCCVII cells was exposed *ex vivo* to ce6-based PDT followed by overnight (16-h) post-incubation. The samples were then prepared for vaccination by insuring that each injection contains 170 *μ*l of wet brei; this was treated with X-rays (60 Gy) and injected peritumorally. The results show that the brei-based PDT vaccine was very effective in inhibiting tumour growth and exhibited potency close to that of cell-based PDT vaccine tested in the same experiment (Figure 8).

## DISCUSSION

It has recently been established that tumour cells treated *in vitro* by PDT can be used for generating potent cancer vaccines (Gollnick *et al*, 2002; Korbelik and Sun, 2006). Injection of such whole-cell PDT vaccine into mice-bearing tumours of the same origin as used for the vaccine generation was shown to produce significant antitumour effect even with models of poorly immunogenic carcinoma (Korbelik and Sun, 2006). The specificity of action against the tumour of vaccine origin was demonstrated by the ineffectiveness against mismatched tumours and the data identify immune rejection mechanisms as responsible for this effect (Gollnick *et al*, 2002; Korbelik and Sun, 2006). A direct demonstration of the engagement of cytotoxic T lymphocytes in the destruction of tumours mediated by PDT vaccine is provided in the present report. High numbers of degranulating CD8^+^ cells were found in lesions regressing after PDT vaccine treatment and much lower numbers in the tumours within the same treatment group that exhibited a poor early responsive (progressing after vaccination). Elevated numbers of degranulating CD8^+^ cells were also found in tumours treated by a regular (direct) PDT treatment (Korbelik and Cooper, 2007).

Although PDT vaccine elicited antitumour immune response is of systemic nature (and cured mice resist tumour re-challenge), the proximity of vaccination site to the treated lesion is relevant for the therapy outcome. While PDT vaccines injected at a distal site were still effective, their impact was inferior to perilesional treatment. This is probably due to the fact that antigen presentation to T cells takes place in the lymph nodes nearest to the vaccine injection site. In case of peritumoral vaccine injection T-cell activation will be centered in tumour-draining lymph nodes, which is a location favourable for T-cell trafficking into the tumour. Thus with the proximal treatment the vaccine effect could be expressed more rapidly than with the distal. Such delay in PDT vaccine-mediated immune activity may prove less important in tumours with slow growth rate.

In our previous studies, we have identified several key parameters for optimal action of PDT vaccine such as the number of PDT-treated tumour cells to be used per vaccination and the concentration for photosensitisers that were employed (Photofrin, BPD) (Korbelik and Sun, 2006). In the present study, ce6 was validated as an effective photosensitiser for generating PDT vaccines and this reveals that various types of photosensitising drugs can be exploited for such role. There are several advantages with using ce6. This established and clinically attested photosensitiser (Spikes, 1990; Sheleg *et al*, 2004) is a simple compound that is water soluble and quickly penetrates into cells, and only a brief incubation (30 min) is sufficient for producing adequate PDT effects. Another advantage of ce6 is its low cost and commercial availability. To stimulate clinically acceptable safety restrictions for the exclusion of the risk from secondary tumour generation, the treatment with a lethal X-ray dose was routinely included as the last step of our protocols for PDT vaccine generation.

This study demonstrates that the potency of PDT vaccine is increased when vaccine cells remain in culture after PDT treatment for an additional time interval (post-incubation protocol) to allow the expression of PDT-induced molecular/biological changes in these cells. Such changes of possible relevance include the progression of apoptotic or necrotic death process associated with the appearance of death signal molecules on the cell surface (Savill and Fadok, 2000; Patel *et al*, 2006; Vandivier *et al*, 2006), and the expression of genes in PDT-treated cells whose products are important immune response mediators; a known example are heat-shock proteins (Gomer *et al*, 1996; Korbelik *et al*, 2005), which appear to have a key role in the action of PDT vaccines (Korbelik and Sun, 2006). The experiments in which PDT vaccine cells were exposed to annexin V and apoptosis inhibitor Z-DEVD (Figure 4 and 5) demonstrate that cell death process and its markers on the cell surface (such as phosphatidylserine) are critical for effective immune processing of these cells. Cell death manipulation is a recognised strategy for augmenting the efficacy of whole-cell cancer vaccines (Copier and Dalgleish, 2006). Our recent findings suggest that the gene encoding serum amyloid P component (SAP), pentraxin protein involved in the disposal of dead cells, is upregulated in the liver and tumour of PDT vaccine treated mice (J Sun and M Korbelik, unpublished results). The process of efferocytosis (removal of dead cells) was recently recognised as a critically important element in the development of PDT-induced tumour immunity (Korbelik, 2006). Around half of PDT vaccine cells collected after the post-incubation protocol were apoptotic and necrosis was evident in about 10% of cells. We have not found that factors released from cells during the post-incubation give additional potency to the PDT vaccine (Figure 3). However, molecules of small molecular weight were lost when concentrating the culture supernatants.

Tumour responses to PDT vaccine showed some variation in the intensity in different experiments. However, in our continuing studies we are now routinely achieving the cures of the two-thirds of PDT vaccine treated tumours; this appears to result from insuring that no tumours are larger than 30 mm^3^ at the time of vaccination. Obviously, PDT vaccines are more effective in eradicating smaller tumour mass but this does not mean that they cannot be employed for therapy of larger lesions. Decreasing the tumour burden by radiotherapy before PDT vaccine treatment allows curing mice with relatively large malignant growth. For instance, the treatment of SCCVII tumours by 20 Gy produced no permanent cures but if this X-ray dose was followed by PDT vaccine application (also not curative when used alone with these tumours) it resulted in 50% tumour cures (M Korbelik, B Stott, J Sun, unpublished results). We are also confident that the potency of PDT vaccines can be further increased by adjuvant immunomodulatory treatment.

This study also demonstrates that tumour tissue can be directly used for the production of PDT vaccine without the need for generating first cultures of single cancer cells. This has very important clinical implications, since tumour tissue surgically removed from the patient can be used for preparing without delay (and with avoiding difficulties/uncertainties in establishing primary cancer cell culture) the PDT vaccine material directly tailored for the individual patient which is acting against tumour antigens existing in that specific tumour.
